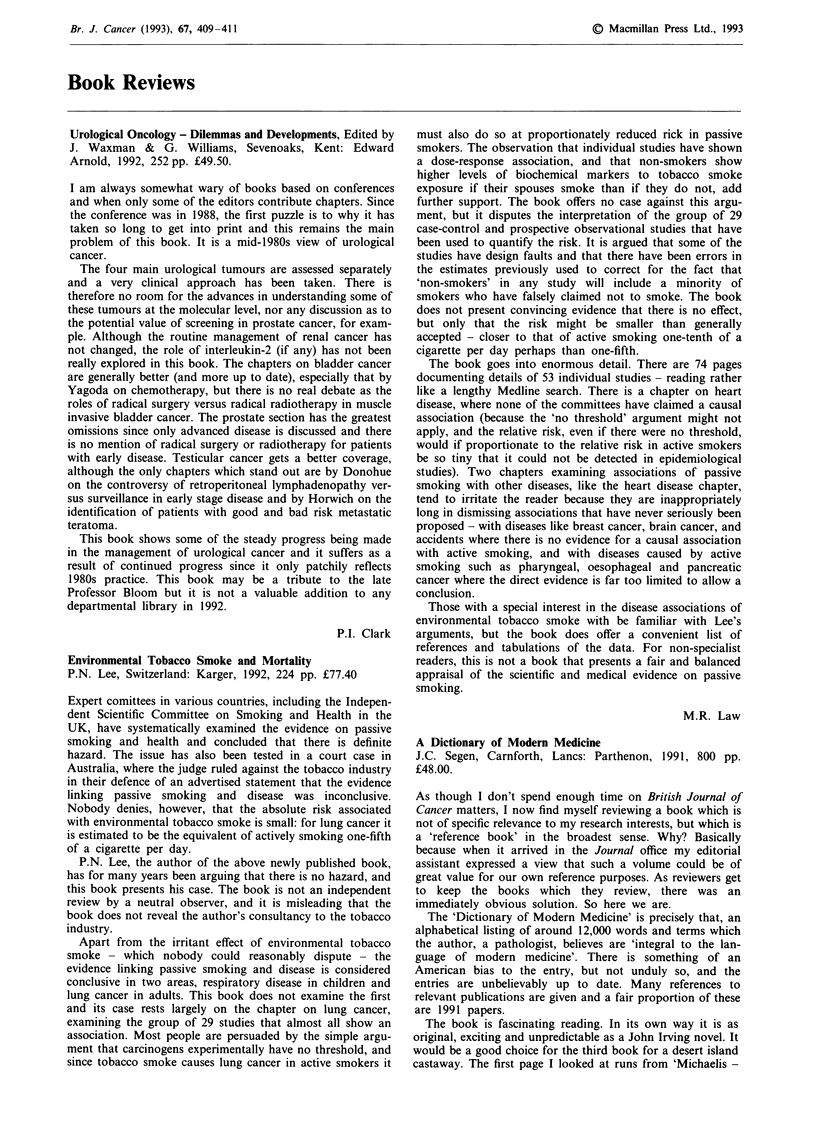# Environmental Tobacco Smoke and Mortality

**Published:** 1993-02

**Authors:** M.R. Law


**Environmental Tobacco Smoke and Mortality**
P.N. Lee, Switzerland: Karger, 1992, 224 pp. £77.40

Expert comittees in various countries, including the Independent Scientific Committee on Smoking and Health in the UK, have systematically examined the evidence on passive smoking and health and concluded that there is definite hazard. The issue has also been tested in a court case in Australia, where the judge ruled against the tobacco industry in their defence of an advertised statement that the evidence linking passive smoking and disease was inconclusive. Nobody denies, however, that the absolute risk associated with environmental tobacco smoke is small: for lung cancer it is estimated to be the equivalent of actively smoking one-fifth of a cigarette per day.

P.N. Lee, the author of the above newly published book, has for many years been arguing that there is no hazard, and this book presents his case. The book is not an independent review by a neutral observer, and it is misleading that the book does not reveal the author's consultancy to the tobacco industry.

Apart from the irritant effect of environmental tobacco smoke – which nobody could reasonably dispute – the evidence linking passive smoking and disease is considered conclusive in two areas, respiratory disease in children and lung cancer in adults. This book does not examine the first and its case rests largely on the chapter on lung cancer, examining the group of 29 studies that almost all show an association. Most people are persuaded by the simple argument that carcinogens experimentally have no threshold, and since tobacco smoke causes lung cancer in active smokers it must also do so at proportionately reduced rick in passive smokers. The observation that individual studies have shown a dose-response association, and that non-smokers show higher levels of biochemical markers to tobacco smoke exposure if their spouses smoke than if they do not, add further support. The book offers no case against this argument, but it disputes the interpretation of the group of 29 case-control and prospective observational studies that have been used to quantify the risk. It is argued that some of the studies have design faults and that there have been errors in the estimates previously used to correct for the fact that 'non-smokers' in any study will include a minority of smokers who have falsely claimed not to smoke. The book does not present convincing evidence that there is no effect, but only that the risk might be smaller than generally accepted – closer to that of active smoking one-tenth of a cigarette per day perhaps than one-fifth.

The book goes into enormous detail. There are 74 pages documenting details of 53 individual studies – reading rather like a lengthy Medline search. There is a chapter on heart disease, where none of the committees have claimed a causal association (because the 'no threshold' argument might not apply, and the relative risk, even if there were no threshold, would if proportionate to the relative risk in active smokers be so tiny that it could not be detected in epidemiological studies). Two chapters examining associations of passive smoking with other diseases, like the heart disease chapter, tend to irritate the reader because they are inappropriately long in dismissing associations that have never seriously been proposed – with diseases like breast cancer, brain cancer, and accidents where there is no evidence for a causal association with active smoking, and with diseases caused by active smoking such as pharyngeal, oesophageal and pancreatic cancer where the direct evidence is far too limited to allow a conclusion.

Those with a special interest in the disease associations of environmental tobacco smoke with be familiar with Lee's arguments, but the book does offer a convenient list of references and tabulations of the data. For non-specialist readers, this is not a book that presents a fair and balanced appraisal of the scientific and medical evidence on passive smoking.

M.R. Law